# Changes in the gut microbiota after hepatitis C virus eradication

**DOI:** 10.1038/s41598-021-03009-0

**Published:** 2021-12-07

**Authors:** Takashi Honda, Masatoshi Ishigami, Kenta Yamamoto, Tomoaki Takeyama, Takanori Ito, Yoji Ishizu, Teiji Kuzuya, Masanao Nakamura, Hiroki Kawashima, Ryoji Miyahara, Tetsuya Ishikawa, Yoshiki Hirooka, Mitsuhiro Fujishiro

**Affiliations:** 1 Department of Gastroenterology and Hepatology, Nagoya University Graduate School of Medicine, 65 Tsuruma-cho, Showa-ku, Nagoya, 466-8550 Japan; 2 Department of Liver, Biliary Tract and Pancreas Diseases, Fujita Health University, Toyoake, Japan; 3 Department of Integrated Health Sciences, Nagoya University Graduate School of Medicine, Nagoya, Japan

**Keywords:** Microbiome, Viral hepatitis

## Abstract

The gut microbiota interacts with infectious diseases and affects host immunity. Liver disease is also reportedly associated with changes in the gut microbiota. To elucidate the changes in the gut microbiota before and after hepatitis C virus (HCV) eradication through direct-acting antiviral (DAA) treatment in patients with chronic hepatitis C (CHC), we investigated 42 samples from 14 patients who received DAA therapy for HCV. Fecal samples were obtained before treatment (Pre), when treatment ended (EOT), and 24 weeks after treatment ended (Post24). The target V3–4 region of the 16S rRNA gene from fecal samples was amplified using the Illumina Miseq sequencing platform. The diversity of the gut microbiota did not significantly differ between Pre, EOT, and Post24. Principal coordinates analysis showed that for each patient, the values at Pre, EOT, and Post24 were concentrated within a small area. The linear discriminant analysis of effect size showed that the relative abundances of *Faecalibacterium* and *Bacillus* increased at EOT, further increased at Post24, and were significantly increased at Post24 compared to Pre. These suggest that changes in the gut microbiota should be considered as among the various effects observed on living organisms after HCV eradication.

## Introduction

Approximately 71 million people worldwide were chronically infected with the hepatitis C virus (HCV) in 2015^1^, and within 20 years, 15%–30% of patients with HCV infections have progressed to cirrhosis, resulting in hepatocellular carcinoma (HCC)^1^. With the recent development of direct-acting antiviral (DAA) therapies, more than 95% of patients with chronic hepatitis C (CHC) can achieve HCV eradication (particularly, viral clearance from an individual's liver, rather than global eradication), which suppresses the progression to cirrhosis and reduces the risk of developing HCC^2^. However, some individuals develop HCC despite HCV eradication, and the risk factors include advanced liver fibrosis, old age, male gender, diabetes, and high fibrosis index based on four factors (FIB-4) index at 24 weeks after the end of treatment^2–4^. Nonetheless, HCC occurs even among people with none of these risk factors, indicating that other causes may be involved in carcinogenesis.

Cells in the human body are outnumbered 10- to 100-fold by microbial cells such as bacteria, fungi, and viruses, including 100 trillion bacteria in the intestines^5,6^. The human gut microbiota stabilizes within approximately 1 year after birth, and thereafter it is affected by factors such as diet, environment, antibiotics, and the use of proton pump inhibitors^7^. The development of next-generation sequencing (NGS) has also made it possible to detect bacteria that are difficult to culture, and changes in the gut microbiota and associated pathologies have been reported to contribute not only to digestive tract diseases but also to liver diseases and diseases in other organs. Recently, it was reported that the gut microbiota interacts with infectious diseases such as enterovirus, HIV, influenza, and hepatitis B and that it also affects host immunity^8^. Liver disease was also reportedly associated with changes in the gut microbiota^9^. Furthermore, an increasing number of studies have shown that lipopolysaccharides, which are a component of the outer membrane of the cell wall of Gram-negative bacteria, flow from the portal vein to the liver, and increased levels of Toll-like receptor ligands promote inflammation and fibrosis in the liver^10–12^. Based on these findings, the gastrointestinal tract and liver are hence considered to have a close relationship, constituting the gut-liver axis^13,14^. Therefore, studies are underway to determine whether the gut microbiota is involved in the progression of liver damage and cirrhosis, as well as the development and promotion of HCC, but results thus far have been inconclusive.

It is possible, then, that there is an interaction between the gut microbiota and the hepatitis C virus. A previous study compared patients with cirrhosis and HCV and those in whom HCV had been eradicated^15^. However, there have been no prospective longitudinal studies on changes in the gut microbiota before and 24 weeks after HCV eradication. Furthermore, whether HCV eradication alleviates dysbiosis in patients with CHC remains controversial^15–18^. Therefore, we conducted this study to elucidate the changes in the gut microbiota before and after HCV eradication through DAA treatment in patients with CHC.

## Results

### Biochemical changes

The patient background information at the start of DAA treatment are presented in Table 1. All patients had CHC, with a median body mass index of 22.0 kg/m^2^. Two patients had diabetes, but their blood glucose control was within the normal range. The median alanine aminotransferase (ALT) value was 22.5 IU/L, and there were no cases of chronic active hepatitis with high ALT fluctuations. Liver biopsy was not performed, but the median FIB-4 index [age × AST/platelet count (10^9^/L) × √ALT] was 2.6. One of the factors for the FIB-4 index was age, and the median patient age in our cohort was 67.0 years.

**Table 1:** Patient characteristics.

	n = 14
Sex (male/female)	2/12
Age (years)	67.0 (57.3–75.8)
BMI (kg/m^2^)	22.0 (19.9–24.6)
Glucose (mg/dL)	94.5 (89.8–112.3)
Total cholesterol (mg/dL)	157.5 (138.3–188.3)
Albumin (g/dL)	4.2 (3.7–4.4)
Total bilirubin (mg/dL)	0.6 (0.6–0.9)
AST (IU/L)	26.5 (23.8–48.8)
ALT (IU/L)	22.5 (15.8–49.3)
γ-glutamyl transpeptidase (IU/L)	18.0 (13.0–31.8)
Platelet count (× 10^4^/mm^3^)	17.2 (13.5–22.2)
Prothrombin time (%)	97.2 (79.7–105.9)
Genotype (1b/1/2b)	11/2/1
HCVRNA (Log IU/mL)	6.3 (5.3–6.8)
FIB-4 index	2.6 (1.1–4.1)
PPI (present/absent)	1/13
DM (present/absent)	2/12
DAA treatment regimens (EBR/GZR)/(OBV/PTV/r)/(SOF/LDV)/GLE/PIB	9/2/2/1
Treatment duration (8 W/12 W)	1/13

*BMI* body mass index, *AST* aspartate aminotransferase, *ALT* alanine aminotransferase, *γGTP* gamma-glutamyl transpeptidase, *HCV* hepatitis C virus, *PPI* proton pump inhibitor, *DM* diabetes mellitus, *EBR* elbasvir, *GZR* grazoprevir, *OBV* ombitasvir, *PTV* paritaprevir, *r* ritonavir, *SOF* sofosbuvir, *LDV* ledipasvir, *GLE* glecaprevir, *PIB* pibrentasvir.

The FIB-4 index of our patients was not high considering their mean age, suggesting that fibrosis had not significantly progressed. Eleven patients had HCV genotype 1b, two had serotype 1, and one had genotype 2b.

Undetectable HCV was confirmed at 24 weeks after the end of the treatment as sustained virologic response (SVR) 24 in all but one patient. One case was HCV RNA-negative up to 16 weeks after the end of the treatment but was virus-positive at 24 weeks after end of the treatment (Supplemental Table 1).

**Figure 1:**
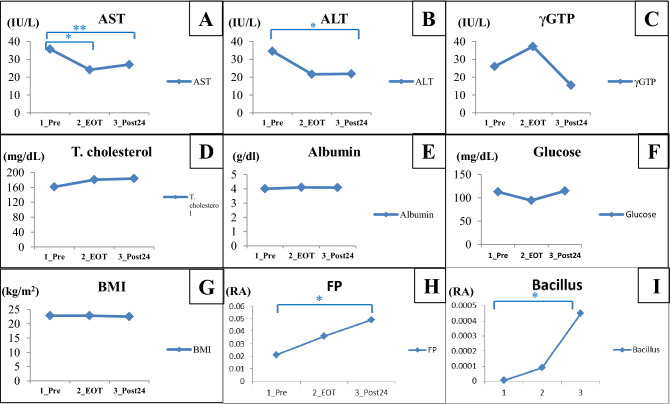
Changes in the gut microbiota and biochemical test results. Comparison between Pre and EOT, Pre and Post24. (**A**) AST, aspartate aminotransferase; (**B**) ALT, alanine aminotransferase; (**C**) γGTP, gamma-glutamyl transpeptidase; (**D**) Total cholesterol, (**E**) Albumin, (**F**) Glucose, (**G**) BMI, body mass index; (**H**) FP, *Faecalibacterium prausnitzii*; (**I**) Bacillus. *P < 0.05, **P < 0.01 Mann–Whitney U test between two points. RA, Relative Abundance.

The biochemical test showed a decrease in AST after the start of treatment; specifically, significantly lower levels were observed at both EOT and Post24 than at Pre (P < 0.01 and P < 0.05, respectively) (Fig. 1A). The ALT levels at Pre and EOT had no significant difference. However, the ALT level at Post24 was significantly lower than that at Pre (P < 0.05) (Fig. 1B). Gamma-glutamyl transpeptidase (γGTP) slightly increased at EOT and decreased at Post24 compared to Pre, but there was no significant difference among each of these time points (Fig. 1C). Total cholesterol and albumin were slightly increased at both EOT and Post24 compared to Pre, but neither difference was significant (Fig. 1D,E). These results indicate that liver inflammation was reduced after treatment, but that there was no significant improvement in liver reserve. The blood glucose levels at EOT and Post24 were not significantly different from that at Pre (Fig. 1G), and BMI likewise did not change (Fig. 1F).

### Changes in gut microbiota

**Figure 2:**
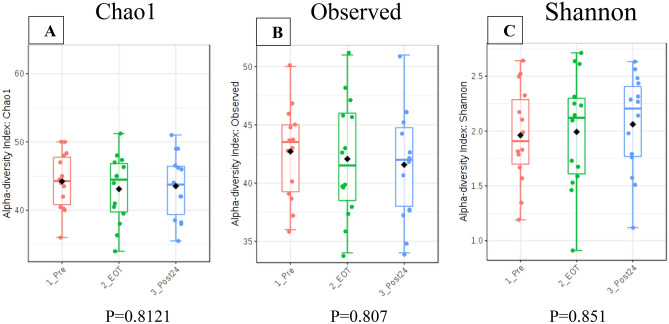
Alpha diversity between Pre, EOT, and Post24 using Chao1 (**A**), Observed (**B**), Shannon (**C**).

During this study, the patient alcohol intake, exercise, and diet which could influence gut microbiota, showed no significant change between Pre and EOT, Post24 (Supplemental Table S1). In all cases, there was no antibiotic use before, during, and up to 24 weeks after treatment. The alpha diversity, which indicates species abundance, had no significant differences between Pre, EOT, and Post24 using the Chao1, Observed, and Shannon Index methods (Fig. 2A–C). Similarly, there were no significant differences between Pre, EOT, and Post24 in terms of beta diversity (Supplementary Fig. S1A,B). In the plan view of the PCoA analysis, the Pre, EOT, and Post24 coordinate plots for each patient were located in the same vicinity (Supplementary Fig. S2).

The results for the Pre, EOT, and Post24 time points at the phylum level are shown for Cases 1 to 14 in Supplementary Fig. S3. In Case 5, Firmicutes was dominant, whereas Bacteroidetes was dominant in the other cases. On average, the proportions of Bacteroidetes and Fusobacteria decreased according to Pre, EOT, and Post24 (Supplementary Fig. S4, green scores), whereas the proportion of Firmicutes and Verrucomicrobia increased (Supplementary Fig. S4, red scores).

**Figure 3:**
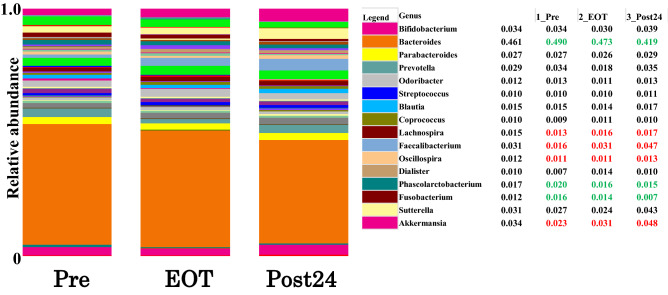
Relative abundance of bacteria is presented at the phylum level. Differences in the microbiota between Pre, EOT, and Post24. Relative abundance of bacteria is presented at the genus level.

Furthermore, when we examined the average of all cases at the genus level, the proportions of the genera *Bacteroides*, *Phascolarctobacterium,* and *Fusobacterium* progressively decreased from Pre to EOT to Post24 (Fig. 3, green numbers), whereas those of the genera *Lachnospira*, *Faecalibacterium*, *Oscillospira*, and *Akkermansia* increased (Fig. 3, red numbers).

**Figure 4:**
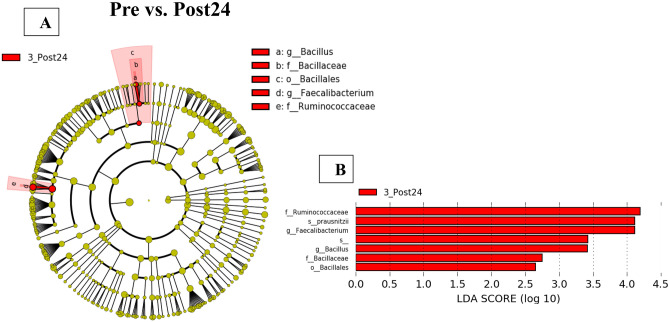
Differences in the microbiota between Pre and Post24. 4 (**A**) Cladogram plotted from linear discriminant analysis of effect size (LEfSe) analysis showing the taxonomic levels. In the circle graph, the objects from the center of the circumference show phylum, class, order (o), family (f), genes (g), species (s), respectively. (**B**) LEfSe plot showing enriched microbiome associated with Post24 (red).

In the multiple comparisons using LEfSe, there was no significant difference in specific gut microbiota between Pre and EOT or between EOT and Post24. However, compared with Pre, Post24 Ruminococcaceae at the family level and *Faecalibacterium* at the genus level were significantly increased. Moreover, Bacillales at the order level, Bacillaceae at the family level and *Bacillus* at the genus level were significantly increased at Post24 (Fig. 4A,B). Furthermore, the relative abundances of *Faecalibacterium* and *Bacillus* were increased at EOT and further increased at Post24 compared to Pre, and the relative abundances of *Faecalibacterium* and *Bacillus* were also significantly increased at Post24 compared to Pre (Fig. 1H,I).

## Discussion

In this study, there was no significant difference between Pre, EOT, and Post24 in terms of either the alpha or beta diversities of the gut microbiome, and the dysbiosis likewise did not vary across the three time points. A previous study also found that patients with cirrhosis treated with DAAs exhibited no significant difference in terms of whether or not HCV had been eradicated; nevertheless, it was not a longitudinal analysis^16^. Similarly, dysbiosis of the gut microbiome in patients with CHC did not exhibit a significant difference before DAA treatment compared to that 3 months after virus eradication^19^. In our PCoA analysis of individual patients, the Pre, EOT, and Post24 values were relatively close to each other, and the change in diversity was relatively small.

In our study, despite the non-alleviation of dysbiosis after HCV eradication, the LEfSe analysis showed that numbers of the *Bacillus* and *Faecalibacterium* genera were increased at Post24 compared to Pre.

*Faecalibacterium prausnitzii*, a butyric-acid producing bacterium with anti-inflammatory effects, reportedly becomes decreased during dysbiosis in the digestive tract, such as in Crohn’s disease^20^. Butyric acid reportedly metabolizes Foxp3 + Treg cells and suppresses inflammation in humans^21^. T cells are also exhausted in persistent viral infections such as CHC and HIV^22^. Moreover, immune status is reportedly normalized by HCV eradication^23^. Based on these facts, it is possible that *Faecalibacterium prausnitzii* recovered its original status along with the recovery of the immune status during the process of HCV eradication. Further studies that include analyses of immunity are therefore needed in the future.

Probiotics containing the genus *Bacillus* reportedly eliminate *Staphylococcus aureus* in the intestines and increase its colonization in the nares^24^. It has been suggested that HCV eradication increases the numbers of *Bacillus* spp. and may thereby reduce infections from bacteria such as *S. aureus*; this process may be related to reverse immunosuppression induced by HCV eradication, similar to the aforementioned increase in *Faecalibacterium prausnitzii*. Further studies on this topic are nevertheless warranted.

The stability of the microbiome in the intestinal tract differs greatly between chronic and acute diseases. In chronic diseases, the microbiome is colonized under conditions of intestinal immunity and remission. This makes it difficult to treat chronic diseases with microbiomes such as fecal microbiota transplant and pre/probiotics. Although there are several reports on SVR and gut microbial changes in patients with cirrhosis, there are insufficient reports on the timing of gut microbial changes in patients with chronic hepatitis C without cirrhosis. Ponziani et al. reported that DAA treatment in patients with cirrhosis C resulted in different gut microbiota before and after 1 year of treatment^16^, indicating that cytokine- and chemokine-induced changes may be important for the changes in the gut microbiome caused by viral eradication. On the other hand, short-term cytokine and chemokine changes and gut microbiota were reported by Pérez-Matute et al^19^. They also showed that cytokine changes were still high at the end of DAA treatment in patients without cirrhosis. Their PCoA plots show more changes in the gut microbiota after 12 weeks than at the end of DAA treatment. From these results, we believe that it is reasonable to assume that the gut microbiota change more after a longer period than at the end of DAA treatment, when cytokine and chemokine activity is still ongoing, even if the virus has been eradicated. These results suggested that changes in gut microbiota are considered to stabilize in about 12 weeks.

The reason *Faecalibacterium prausnitzii* and *Bacillus* showed no significant increase at EOT compared to Pre is that although all 14 patients were virus negative at EOT, eight patients had negative results 4 weeks before EOT, and changes in the intestinal flora may not have been completed yet during EOT. By Post24, at least 12 weeks had passed because the virus was negative at 12 weeks after the end of treatment in all patients. Therefore, Post24 is considered to reflect adequately the extent of the changes in the gut microbiota.

The effects of HCV eradication on the gut microbiota in liver cirrhosis have been previously reported. *Enterobacteriaceae*, *Enterococcus*, and *Staphylococcus* decrease after treatment, but intestinal permeability and inflammation do not change^15^. The present study excluded patients with cirrhosis and examined only those with CHC. Therefore, the *Clostridiales* genus did not decrease and the *Streptococcus* and *Lactobacillus* genera did not increase, whereas the *Bacillus* and *Faecalibacterium prausnitzii* increased. The study mentioned above suggested that virus control in patients with liver cirrhosis may alter the gut microbiota associated with improved liver reserve and liver fibrosis. By contrast, our study examined only patients with chronic hepatitis to assess the direct effects of virus control. Compared to the aforementioned study that included patients with advanced liver fibrosis or cirrhosis, our study did not demonstrate significantly improved hepatic reserve function, indicating a direct effect of changes in the gut microbiome after HCV eradication.

Dysbiosis of the gut microbiome reportedly worsens as patients progress from chronic hepatitis to liver cirrhosis, with intestinal *Clostridiales* decreasing, and *Streptococcus* and *Lactobacillus* increasing with the progression of chronic hepatitis C to cirrhosis^25^. Our study only examined patients with CHC, and therefore these aforementioned changes in the gut microbiome were not observed.

There are several different types of hepatitis C antivirals. We performed a clinically based study, and our patients included those who have received different types of treatment that were covered by health insurance in Japan. However, the DAAs that we used are very liver-specific and act directly on the infected HCV. In addition, the duration of treatment differs by only a few weeks, with most drugs achieving negative viral levels early. The difference in the duration of treatment is based on the different periods required to maintain viral eradication to prevent a recurrence. From these results, we believe that changes in the human body due to the absence of the virus and the virus-bacteria relationship are important factors. Therefore, we believe that the effect of drug differences on the results of this study is minimal.

Most of the patients did not have significant lifestyle changes after starting treatment. This may be because many patients had already established their lifestyles. Therefore, we believe that this study had little influence on changes of gut microbiota in individual patients due to lifestyle changes, because of intra-personal comparisons.

A study from Japan compared fecal samples from elderly, matched urban residents with those from long-lived suburban residents^26^. According to this study, residents of suburban areas with high longevity had a higher relative abundance of Firmicutes, especially butyrate-producing Roseburia spp. Case 5 was 85 years old, the oldest of the 14 cases, and according to the questionnaire, the relative abundance of Firmicutes was higher in patients with a diet that included Japanese food and vegetables and with a high frequency of exercise. The abundance of Roseburia was also higher than in other patients (0.00029% vs. 0.00015%). These characteristics may be related to the gut microbiota found in long-lived individuals.

The limitation of this study is the lack of healthy controls and the small number of enrolled patients with CHC. For a better understanding of changes in the gut microbiota, it may be better to use healthy control groups and compare them to patients with HCV, but it is ethically difficult to establish a true control group of older and healthy control patients who are also administered with DAA similar to patients with CHC. It is also difficult to accumulate healthy people with the same median age (67 years) as our cohort of patients with CHC. In addition, the gut microbiota of each patient is quite different from each other. Therefore, we compared the changes over time in the same patients. For this reason, it had not been possible to determine whether the gut microbiota was similar in patients with CHC before treatment and in healthy individuals. Further studies are therefore needed in this regard.

In this study, we observed the changes in the gut microbiota due to HCV control in CHC. The diversity of the gut microbiota did not significantly change in patients with CHC after HCV eradication. However, the relative abundances of *Bacillus* and *Faecalibacterium prausnitzii* were significantly increased in patients 24 weeks after treatment (Post24) compared to those before treatment (Pre). This suggests that changes in the gut microbiota after HCV eradication have secondary effects that should be considered.

## Methods

### Patients and samples

This prospective longitudinal study involved patients with CHC who have been treated with DAAs at the Department of Gastroenterology and Hepatology of the Nagoya University Hospital. Patients were given a sufficient explanation of the study and were then asked to provide written consent to participate, and written informed consent was obtained from each patient. The study protocol was approved by the Ethics Committee of Nagoya University School of Medicine (2016-0428) and was conducted following the Declaration of Helsinki (1975).

DAAs were administered for the treatment of hepatitis C, and stool samples were collected before DAA treatment (Pre), at the end of treatment (EOT), and at 24 weeks after the end of treatment (Post24). At each point, the changes in diet, exercise, and alcohol intake, which could influence gut microbiota, were monitored. We use a self-administered questionnaire to survey their lifestyle, including diet, exercise, and alcohol intake. Blood was collected every 4 weeks from the start of treatment for biochemical tests to monitor the side effects of regular DAA treatment, and serum was stored at − 80 °C.

Among the patients treated with DAAs, three received steroids, three had a history of treatment for HCC, and one was excluded because of cirrhosis. Finally, 14 patients with CHC were examined in this study.

### Microbiota analyses

All procedures were performed according to the protocol of the Human Microbiome Project. DNA was extracted from patient stool using the DNeasy PowerSoil DNA Isolation Kit (Qiagen, Hilden, Germany), and was purified to 5 ng/µL. Amplicon PCR specifically targeted the V3–4 region of 16S rRNA (forward primer: 5'-TCGTCGGCAGCGTCAGATGTGTATAAGAGACAGCCTACGGGNGGCWGCAG-3', reverse primer: 5'-GTCTCGTGGGCTCGGAGATGTGTATAAGAGACAGGACTACHVGGGTATCTAATCC-3'; 95 °C). Initial denaturation was performed at 95 °C for 3 min, followed by 25 cycles of denaturation at 95 °C for 30 s, primer annealing at 55 °C for 30 s, extension at 72 °C for 30 s, and final elongation at 72 °C for 5 min. The Amplicon PCR product was 550 bp in length and was cleaned up using AMPureXP beads (Beckman Coulter, Brea, CA, USA). Next, a Nextera XT index kit (Illumina, San Diego, CA, USA) was used for index PCR. Initial denaturation was conducted at 95 °C for 3 min, followed by 8 cycles of denaturation at 95 °C for 30 s, primer annealing at 55 °C for 30 s, extension at 72 °C for 30 s, and final elongation at 72 °C for 5 min. Subsequently, the DNA was purified using the same beads as described above. After a 4 nM cDNA library was created from each sample, a 20% spike of Phix was added and paired-end reads were performed with 600 cycles of 2 × 300 reads using the MiSeq Reagent Kit V3 (Illumina) at a final concentration of 8 pM. Sequences were determined using the Illumina Miseq sequencer (Illumina). The Quantitative Insights into Microbial Ecology (QIIME1.9.1) software (http://qiime.org) was used for the subsequent analysis of 16S rRNA gene sequence data and Greengenes 13_8 was used as an operational taxonomic unit (OTU) database^27^. After the chimera check, the OTU was determined and a secondary analysis was performed using the linear discriminant analysis of effect size (LEfSe), the Greengenes13_8 database provided by the Galaxy module (http://huttenhower.sph.harvard.edu/galaxy/) that explains differences between groups by coupling standard tests for statistical significance with additional tests encoding biological consistency and effect relevance, and the Microbiome Analyst (https://www.microbiomeanalyst.ca) for comprehensive statistical, functional, and meta-analysis of microbiome data^28^.

### Statistical analyses

The categorical chi-square test or Fisher’s exact test was used in comparing two groups. In the case of a continuous variable, the Student’s t-test or the Mann–Whitney U test was performed as necessary. The alpha diversity of the gut microbiota, which indicates species richness, was analyzed using the Chao1, Observed, and Shannon methods, and the beta diversity was analyzed using principal coordinates analysis (PCoA).

## Supplementary Information

Supplementary Information 1.

Supplementary Information 2.

## Abbreviations

HCV: Hepatitis C virus
HCC: Hepatocellular carcinoma
DAA: Direct-acting antiviral
CHC: Chronic hepatitis C
NGS: Next-generation sequencing
EOT: End of treatment
OUT: Operational taxonomic unit
LEfSe: Linear discriminant analysis of effect size
PCoA: Principal coordinates analysis
AST: Alanine aminotransferase
ALT: Alanine aminotransferase
