# Supplementary material for: Changes in the gut microbiota after hepatitis C virus eradication
Source: Sci Rep. 2021 Dec 7;11:23568. doi: 10.1038/s41598-021-03009-0 (PMC8651745; doi:10.1038/s41598-021-03009-0)
Supplement: Supplementary file 1 — Supplementary Information 1. [file 41598_2021_3009_MOESM1_ESM.docx]

Supplemental Table1

Questionnaire on lifestyle and detection of HCV-RNA at three time points

|  | | | | n | Pre | n | EOT | p-value | n | Post24 | p-value |
| --- | --- | --- | --- | --- | --- | --- | --- | --- | --- | --- | --- |
|  | | | | Questionnaire on alcohol and exercise lifestyle | | | | | | |  |
| Alcohol (0/<60 g/day) | | | | 11 | 11/0 | 12 | 12/0 | 1.0000 | 11 | 11/0 | 1.0000 |
| Exercise habits once a week (no/yes) | | | | 9 | 7/2 | 12 | 9/3 | 0.6471 | 11 | 7/4 | 0.4257 |
|  |  |  | Questionnaire on dietary lifestyle * | | | | | | | | |
| Main protein intake (meat/fish) | | | | 11 | 4/7 | 11 | 4/7 | 0.6703 | 9 | 4/5 | 0.5350 |
| Vegetables  (enough/not enough) | | | | 9 | 7/2 | 12 | 9/3 | 0.6471 | 11 | 9/2 | 0.6254 |
| Style (Japanese/Western) | | | | 10 | 6/4 | 11 | 6/5 | 0.5750 | 11 | 7/4 | 0.6084 |
| HCV-RNA, (detected/undetected) | | | | 14 | 14/0 | 14 | 0/14 | <0.001 | 14 | 1/13 | <0.001 |

EOT, end of treatment; HCV, hepatitis C virus; SVR, sustained virological response.

* Patients were given a self-report questionnaire to choose from two options for a more suitable dietary lifestyle.

Patients were allowed to choose not to answer questions about their lifestyle due to privacy or their wishes. Therefore, some patients have not been able to complete the questionnaire.
