# Supplementary material for: Changes in the gut microbiota after hepatitis C virus eradication
Source: Sci Rep. 2021 Dec 7;11:23568. doi: 10.1038/s41598-021-03009-0 (PMC8651745; doi:10.1038/s41598-021-03009-0)
Supplement: Supplementary file 2 — Supplementary Information 2. [file 41598_2021_3009_MOESM2_ESM.pdf]

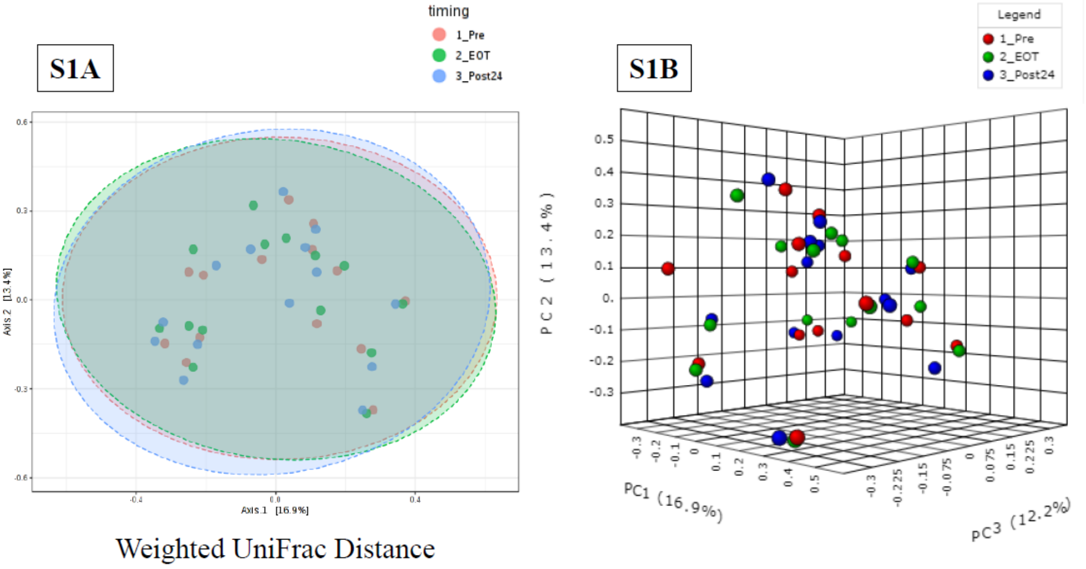

Weighted UniFrac Distance

Supplementary Fig.1

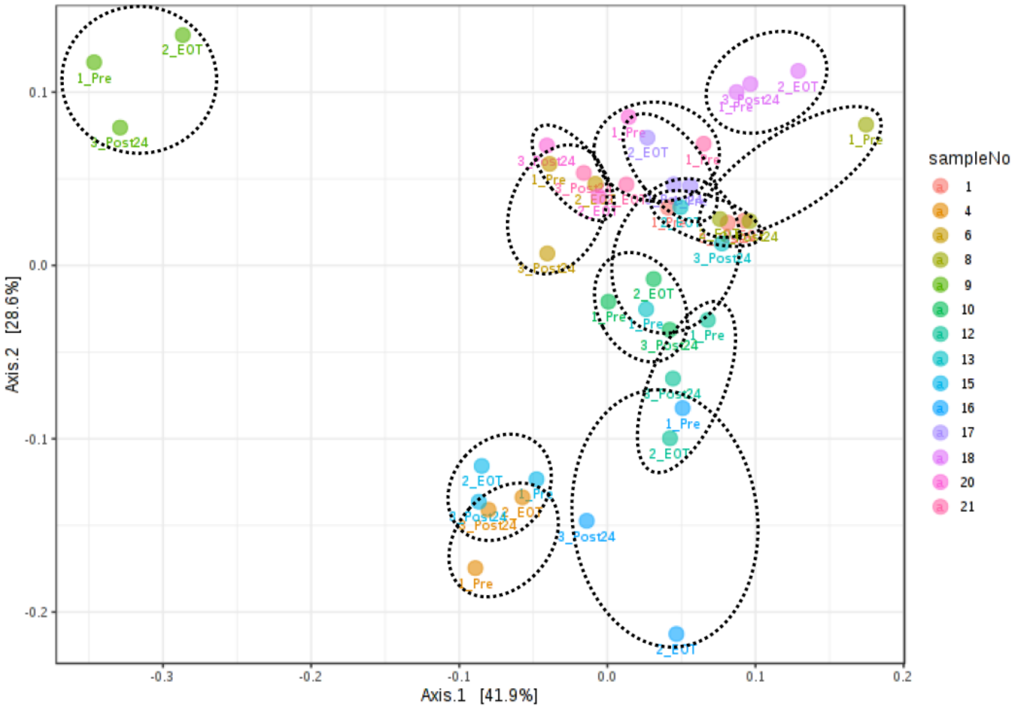

Supplementary Fig.2

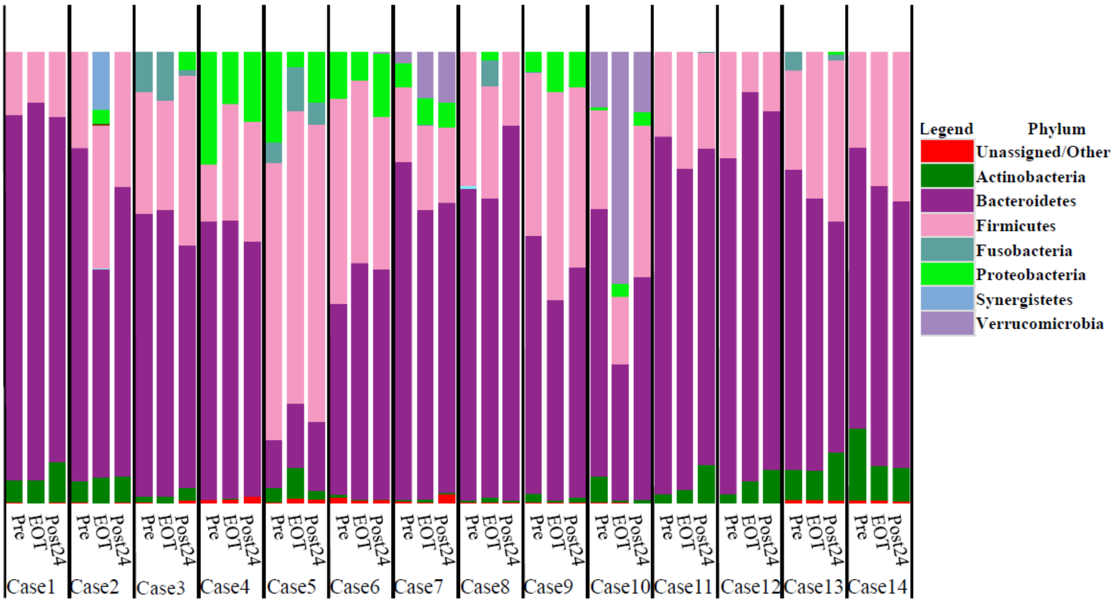

Supplementary Fig.3

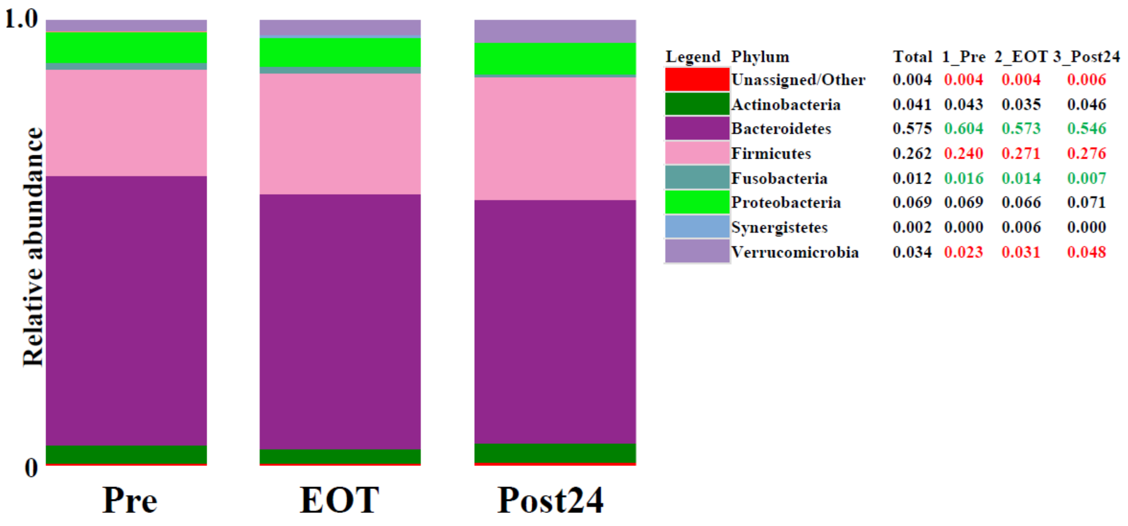

Supplementary Fig.4

Supplementary Fig.1 Beta diversity principal coordinates analysis (PCoA). Clustering of Pre (red), EOT (green), and Post24 (blue). (S1A) PCoA of Pre, EOT, and Post24 (S1B) Each dot represents a subject in the graphs and the distance between the dots is proportional to the similarity in the microbial abundance pattern.

Supplementary Fig.2 Principal coordinate analysis (PCoA) for the weighted UniFrac distance of microbiome samples. The Pre, EOT, and Post24 coordinate plots for each patient (same color) were enclosed with dash line and located in the same vicinity.

Supplementary Fig.3 Differences in the microbiota between Pre, EOT, and Post24 in

each patient.

Supplementary Fig.4 Relative abundance of bacteria is presented at the phylum level.

Differences in the microbiota between Pre, EOT, and Post24.
